# BCR-ABL fusion genes and laboratory findings in patients with chronic myeloid leukemia in northeast Iran

**DOI:** 10.22088/cjim.9.1.65

**Published:** 2018

**Authors:** Hossein Ayatollahi, Mohammad Reza Keramati, Abbas shirdel, Mohammad Mehdi Kooshyar, Majid Raiszadeh, Sepideh Shakeri, Mohammad Hadi Sadeghian

**Affiliations:** 1 Cancer Molecular Pathology Research Center, Mashhad University of Medical Sciences, Mashhad, Iran; 2 Department of Internal Medicine, Faculty of Medicine, Mashhad University of Medical Sciences, Mashhad, Iran.; 3 Department of Hematology and Blood Bank, Faculty of Medicine, Mashhad University of Medical Sciences, Mashhad, Iran

**Keywords:** BCR-ABL, RT-PCR, chronic myeloid leukemia, Iran

## Abstract

**Background:**

A specific chromosomal abnormality, the Philadelphia chromosome (BCR-ABL fusion), is present in all patients with chronic myeloid leukemia (CML). The b2a2 and b3a2 fusion mRNAs encode p210 fusion protein p210 and e1a2 encode p190. The aim of this study was to evaluate the frequency of BCR-ABL fusion transcript variants in Northeast of Iranian CML patients and to compare the laboratory results of our patients.

**Methods:**

This study was conducted in 85 peripheral blood and bone marrow samples of CML patients. Ribonucleic acid (RNA) was extracted by a commercial kit, RT- PCR for identifying BCR-ABL fusions was carried out by using designed primers and the PCR products were electrophoresed in agarose gels. Finally, statistical analysis was performed for variant frequency identification and their comparison was performed.

**Results:**

All patients examined were positive for BCR/ABL rearrangement. Fusion of b3a2 was detected in 53 (62.35%) patients, b2a2 in 25 (29.41), e1a2 in 1 (1.17%) and coexpression of b3a2 and e1a2 in 6 (7.05%) patients. There were significant differences between the mean age in patients with b3a2 positive ( 44.07 years) and in b3a2 negative group (50.35 years) however, no significant differences were seen between sex and b2a2 (P=0.61), b3a2 (P=0.79) and e1a2 (P=0.20).

**Conclusions:**

This study showed higher frequency b3a2 than b2a2 and e1a2 transcripts in CML patients in Northeast Iran and there was no association between e1a2 transcripts frequencies and monocytosis in peripheral blood.

CML (chronic myeloid leukemia) or chronic granulocytic leukaemia is one of the myeloproliferative neoplasms (MPN) characterized by proliferation of a pluripotential stem cell that can differentiate along the granulocytic lineage and is consistently associated with the *BCR-ABL1* fusion gene (1). It accounts about 15% of all leukemia cases (2). The age-adjusted incidence rate is approximately 2.0 cases per 100000 people every year that increase with age, average age, average age at diagnosis is around 65, and is higher in men than in women (3). CML is typically associated with a balanced reciprocal translocation between the long arms of chromosomes 9 and 22 or t(9;22) resulting to a shortened chromosome 22 (Philadelphia chromosome). This translocation is named Philadelphia chromosome (Ph), and was first reported in 1960 by Peter Nowell and David Hungerford in the chromosomes from cultured blood cells of two patients with CML (4). The Ph chromosome (BCR-ABL fusion gene) is formed by fusion of the 3´ sequences from *ABL1 *(Abelson) gene at 9q34 to the 5´ portion of the *BCR* (breakpoint cluster region) gene sequences at 22q11. The product of this fusion *BCR-ABL *gene is a constitutively active protein tyrosine kinase, p210 *BCR-ABL, *that promotes cellular proliferation and suppresses apoptosis. 

BCR-ABL kinase activity is critical to the development of CML (5). Although BCR-ABL fusion gene can be detected in all CML patients at the molecular level; it is not limited to CML. This balanced translocation may also be seen in other malignancies such as acute lymphoblastic leukemia and occasionally in newly diagnosed acute myeloid leukemia (AML) (6-9). 

The *BCR *gene has 23 exons (e1 to e23) and the *ABL *gene has 12 exons (1b; 1a; a2 to a11). The breakpoints within the ABL gene (at 9q34) occur in a nearly constant **region**. In CML, breakpoint in this gene is variable over a region of about 300 kb at the 5' end of the ABL gene and often between the two alternative exons 1b and 1a (always 5' of exon 2). In CML, the breakpoints within the BCR gene are localized to one of three main regions. The commonest is the major breakpoint cluster region, (M-BCR), a cluster of 5.8 kb within BCR exons 12 and 16, also called b1 to b5. Generally, the break occurs within introns located between exons b2 and b3 or b3 and b4 with the exon a2 forming the fusion gene b2a2 or b3a2, respectively. A 210-kd chimeric protein is derived from this mRNA (P210). The second groups of breakpoints occur in the 54.4-kb region between the alternative *BCR *exons e2' and e2 region, termed the minor breakpoint cluster region (m-*bcr*). The resultant e1a2 mRNA is translated into a 190-kd protein (P190). Due to alternative splicing, a small amount of P190 may be found in many CML patients. The third breakpoint cluster region (µ-*bcr*) is identified between BCR exons 19 and 20 (downstream of exon 19). The resultant e19a2 mRNA is a 230-kd fusion protein (P230) (10-12).

Detection of BCR-ABL1 fusion transcript variants may have clinical importance. De Lemos et al. showed that B2A2 may be more sensitive to imatinib than b3a2 and concluded that CML patients who express the B2A2 may have a better prognosis (13). There are some studies about relationship between ela2 and prominent monocytosis (14); and high platelet counts and also marked splenomegaly have been reported in CML patients with coexpression of the p190/p210 (15). This study was done to determine the frequency of BCR-ABL fusion genes and laboratory findings of patients with chronic myeloid leukemia in northeast Iran. 

## Methods

This cross-sectional study was performed on 85 cases of CML referred by hematologists to molecular pathology laboratory of Ghaem Hospital, Mashhad, Iran in 2012-2013. The study was approved by local ethics committee and also financially supported by the Research Vice Chancellery of Mashhad University of Medical Sciences. All patients had clinical, complete blood count (CBC), peripheral blood smear (PBS) and bone marrow (BM) features of CML such as splenomegaly, leukocytosis, eosinophilia, basophilia, neutrophilia, 1-10% blasts in PBS or BM. Diagnosis of CML was done according to WHO criteria (1). Patients with incomplete criteria for diagnosis of CML were excluded. For each patient, five to ten milliliter anticoagulated peripheral blood (with ethylenediaminetetraacetic acid, EDTA-K2) or 0.5-1 ml of bone marrow aspiration was drawn and then RNA was extracted by RNeasy Mini kit (Qiagen, Valencia, CA) according to the manufacturer's instruction. 

RNA concentration was determined applying the Thermo Scientific NanoDrop 2000 Spectrophotometer and specimens with low RNA content (<20 ng/µL) were excluded from the study. After that, cDNA was synthesized by cDNA synthesis kit (Fermentas UAB, Lithuania). Finally, PCR for BCR-ABL was achieved by nested PCR with a set of external primers (A& B) and internal primers (C&D) as follows: 

**BCR-ABL p190 fusion gene,**

BCR-e1-A 5´-GACTGCAGCTCCAATGAGAAC- 3 ´,

 ABL-a3-B 5´-GTTTGGGCTTCACACCATTCC-3´, 

BCR-e1-C 5´-CAGAACTCGCAACAGTCCTTC-3´,

 BCR-e3-D 5´-CAGAACTCGCAACAGTCCTTC-3´

**BCR-ABL p210 fusion gene,**

BCR-b1-A 5´-GAAGTGTTTCAGAAGCTTCTCC-3´, 

ABL-a3-B 5´-GTTTGGGCTTCACACCATTCC-3´,

 BCR-b2-C 5´-CAGATGCTGACCAACTCGTGT-3´

ABL-a3-D 5´-TTCCCCATTGTGATTATAGCCTA-3´

**ABL** **Control gene:** ABL-F 5´-CCT TCA GCG GCC AGT AGC-3´

ABL-R 5´-GGA CAC AGG CCC ATG GTA C-3´

PCR was performed in total volume of 25µL by adding 2-3 µL of cDNA, 1U/ µL Taq enzyme, 200µM dNTP, 2.5 Mm Mgcl2, 400 nmol of each primers, 20 mM Tris HCL , 50 mM KCL (pH=8.3) and distilled water. For the second round, PCR 1 µL of first round PCR products were added. 

cDNA synthesized from K562 cells (b3a2) and KCL22 for b2a2 cells type and ALL/MIK for p190 e1a2 were used as positive controls and sterile water was used as negative controls. In this study, we used ABL gene as a control gene. The tubes were placed into Applied Biosystem (ABI) Veriti Thermal Cycler and PCR was started with 30 seconds at 94^o^C for the first step and then 35 cycles was run as follows: 30 seconds at 94°C, 60 seconds at 65°C, 60 seconds at 72°C and final extension was 10 min at 72^o^C. Products were electrophoresed on a 2% agaros gel with robust DNA stain. Bands of 521 bp ,417 bp, 342 and 340 bp were observed for e1-a2, b3-a2,b2-a2 and normal ABL, respectively, for first round PCR and bands of 381 bp ,360 bp ,285 bp and 282 bp were detected e1-a2, b3-a2,b2-a2 and normal ABL, respectively, in second nested PCR, respectively. Statistical analysis: RT-PCR (PCR results) and other laboratory findings were analyzed with SPSS Version 11.5. We used Pearson’s chi-square test for comparison of categorical variables and independent sample t-test and Mann-Whitney test for continuous variables between the case and the control groups. A p-value below 0.05 was considered significant.

## Results

P210 BCR/ABL rearrangements (b2a2 and b3a2) were more common than P190 (e1a2). Fusion of b3a2 was detected in 53 (62.35%) patients, b2a2 in 25 (29.41**%**), e1a2 in 1 (1.17%) and both b3a2 and e1a2 in 6 patients (7.05%). Patients' age range and mean age (±SD) were 21 to 77 years and 45.89±15.60 years, respectively. 

There was no significant difference between b2a2 (P=0.095) positive and negative groups and also between e1a2 (P=0.48) positive and negative groups for mean age of patients. 

The mean age (±SD) in patients with b3a2 positive was 44.07(±SD) years and in b3a2 negative group was 50.35 (±SD)years and Mann-Whitney test showed a significant difference between these two groups (P=0.046). 

**Table 1:** Comparing CBC, peripheral blood smear and biochemical findings in b3a2, b2a2 and e1a2 positive and negative patients

**e1a2**			**b2a2**				**b3a2**		**Variables**	
**Pvalue**	**Positive**	**Negative**	**Pvalue**	**Positive**	**Negative**	**Pvalue**		**Positive**	**Negative**	
	**Mean** **±** **SD**	**Mean** **±** **SD**		**Mean** **±** **SD**	**Mean** **±** **SD**			**Mean** **±** **SD**	**Mean** **±** **SD**	
0.22	3.2±0.8	3.5±0.7	0.78	3.5±0.6	3.5±0.7	0.94		3.5±0.7	3.5±0.6	RBC x 10^12^ /L
0.35	153.9±72.1	131.9±58.8	0.89	135.1±43.7	133.1±65.7	0.98		133.6±66.2	133.9±43.2	WBC x 10^9^ /L
0.14	518.1±623.6	368.3±206.2	0.39	343.1±233.2	396.3±273.0	0.26		401.7±272.1	333.0±234.3	PLT x 10^9^ /L
0.29	29.4±6.8	31.8±5.7	0.35	32.5±5.1	31.2±6.1	0.59		31.3±6.0	32.1±5.4	HCT (L/L)
0.20	9.4±1.9	10.3±1.8	0.15	10.6±1.5	10.0±1.9	0.36		10.1±1.8	10.5±1.7	HGB (mg/dL)
0.67	90.8±5.2	89.4±8.3	0.09	91.8±9.7	88.6±7.2	0.10		88.6±7.2	91.7±9.5	MCV (fL)
0.99	29.2±3.0	29.3±4.1	0.11	30.3±4.7	28.8±3.7	0.16		28.8±3.7	30.2±4.7	MCH (Pg)
0.77	32.2±2.85	32.5±2.8	0.39	32.9±2.3	32.3±2.9	0.53		32.3±2.9	32.8±2.3	MCHC(mg/dL)
0.06	8.9 ±7.3	3.6±4.1	0.56	3.6±4.2	4.3±5.7	0.99		4.1±5.5	4.1±4.8	Blast %
0.84	6.4±3.1	6.6±3.1	0.38	6.2±3.5	6.8±2.9	0.25		6.9±2.9	6.0±3.4	Eosinphils %
0.14	5.5±2.1	**7.7±3.9**	0.14	6.6±3.4	7.9±3.9	0.09		8.0±3.9	6.5±3.4	Basophils %
0.26	39.7±7.8	44.2±10.3	0.01	48.5±10.0	41.8±9.7	0.05		42.1±9.5	48.7±9.8	Neutrophils %
0.93	25.2±11.7	25.0±6.6	0.11	23.2±7.2	25.8±6.9	0.07		26.0±6.7	22.8±7.4	Myelocyte %
0.95	10.4±12.9	6.0±3.9	0.54	5.8±3.2	6.6±5.9	0.82		6.1±4.4	7.0±6.8	Monocytes %
0.48	41.8±14.9	46.2±15.7	0.47	36.5±20.4	49.8±43.2	0.47		49.8±43.2	36.5±20.4	Urea (mg/dL)
0.22	3.2±0.8	3.5±0.7	0.31	1.0±0.5	1.6±1.2	0.31		1.6±1.25	1.0±0.5	Creatinine(mg/dL)
0.35	153.9±72.1	131.9±58.8	0.96	141.0±5.8	140.8±4.4	0.96		140.8±4.4	141.0±5.85	Sodium
0.14	518.1±623.6	368.3±206.2	0.70	4.2±0.4	4.3±0.6	0.70		4.3±0.6	4.2±0.46	Potassium
0.29	29.4±6.8	31.8±5.7	0.99	9.4±0.98	9.4±0.2	0.99		9.4±0.2	9.4±0.98	Calcium
0.20	9.4±1.9	10.3±1.8	0.09	8.7±3.3	5.7±1.3	0.09		5.7±1.3	8.7±3.3	Uric acid
0.67	90.8±5.2	89.4±8.3	0.96	2315.0±1926.0	2360.4±964.1	0.96		2360.4±964.1	2315.0±1926.0	LDH
0.99	29.2±3.0	29.3±4.1	0.25	33.2±15.5	19.0±14.7	0.25		19.0±14.7	33.2±15.5	AST
0.77	32.2±2.85	32.5±2.8	0.35	23.6±14.6	14.6±3.2	0.35		14.6±3.2	23.6±14.6	ALT

Forty-one (48.2%) patients were male and 44 (51.8%) patients were females. Pearson’s chi-square test showed no significant difference between sex and b2a2 (P=0.61), b3a2 (P=0.79) and e1a2 (P=0.20). Splenomegaly was present in 52 (61.2%) patients and hepatomegaly in 24 (28.2%). Although splenomegaly and hepatomegaly were more common in the p210 positive group than the negative group, these differences were not significant (P=0.06% and P=0.08%, respectively) Nine (10.6%) patients (5 males and 4 females) were in accelerated phase and 76 (89.4%) patients in chronic phase of CML. 

There was not any significant differences between different phases of CML (accelerated or chronic) and kind of BCR/ABL fusion (P=0.22), sex (P=0.64) and patients’ age (P=0.15); however, patients’ age in accelerated phase (52.89±16.09 years) was more than the chronic phase (45.07±15.44 years). 

Table 1 shows the laboratory findings of CML patients and compare the positive and negative BCR/ABL fusion results. Neutrophil precentage in b3a2, b2a2 and e1a2 positive groups was higher than the negative groups.

## Discussion

In our study, b3a2 transcripts were more frequent than b2a2 (62.35% vs 29.41%). This finding is concordant with the studies of Todoric-Zivanovic et al. (16), Bennour et al. (17) and Anand et al. (18) nevertheless by contrast, in the studies of Arana-Trejo et al. (15), Rosas-Cabral et al. (19) and Muddathir et al. (20). In a study by Todoric-Zivanovic et al. in Siberia b3a2 was reported in 74%, b2a2 in 25.0% and b3a2 / e1a2 in one (1%) (16) patient. Another study by Bennour et al. in Tunisia, CML patients showed that more than half of them had b3a2 fusion transcript (64 %), almost one third (36 %) b2a2 transcript, nonetheless, coexpression of b3a2 and b2a2 was not detected in any patients. (17). Anand et al. (18) demonstrated b3a2 in 67%, b2a2 in 29% , b3a2/ b2a2 in 4%, b3a2/e19a2 in 0.5% and b2a2/e19a2 in 0.5% in India. Arana-Trejo et al. (15) found b3a2 in 35%, b2a2 in 48%, and both b3a2 and b2a2 in 3% in Mexico. Rosas-Cabral et al. (19) detected b3a2 bcr-abl in 28% cases, b2a2 in 59% cases, and 13% with both b3a2 and b2a2 in Spanish patients; while Muddathir et al. (20) reported b3a2 bcr-abl transcripts in 21 patients, b2a2 in 6 cases, and 8 patients with both b3a2/b2a2 in Sudanese patients.

Coexpression of different transcriptants may occur due to *change in* the *phenotype* of   the leukemic cells and also due to alternative splicing of mRNA. The co-expressed p190/p210 transcript frequencies in our CML (7.05%) patients were similar in Mexican population (5%) and were less frequent than the Sudanese population (17.4%) (15, 19). Furthermore, marked splenomegaly and high platelet counts in coexpressed p190/ p210 patients were reported by Arana-Trejo (15) although we did not find a significant difference for p190/p210 transcripts and platelet count (p=0.12) and also in the relationship between p190/p210 transcripts and splenomegaly (p=0.13). Similar to our study, Yaghmaie et al. did not find any significant differences between clinical findings in CML patients and BCR-ABL breakpoints (23).

In our study, the patients with b2a2 were older than the patients with b3a2 transcripts, yet there was not significance (p=0.16). *This finding was statistically significant in the study by* Bennour et al. (17). Male to female ratio in our study was 29/30 for b3a2, 11/14 for b2a2 and 5/2 for e1a2; and Goh et al. (21) and Bennour et al. (17). Our study did not reveal the significant differences between sex and b2a2 (p=0.61), b3a2 (p=0.79) or e1a2 (p=0.20). Despite that, high male to female ratio for b2a2 (15.8) and low ratio for b2a2 (5/13) have been reported by Osman et al. (22).

Inspite of the association between P190 with peripheral blood monocytosis in CML has been reported (14), our results did not show any relationship between e1a2 transcript frequencies and (p=0.95) and absolute monocyte count (p=0.58). Notwithstanding, our results showed higher neutrophil percentage in the b3a2 and b2a2 positive groups, in the negative groups absolute neutrophilic count (p=0.27 for b3a2 and p=0.25 for b2a2) was not significant. Small size population was one of present study’s limitations and it is better to to conduct it in large population; another limitation was the lack of access to patients for follow-up and calculate survival markers to determine BCR-ABL fusion transcribe.

In conclusion,** t**he results of our study showed higher frequency b3a2 than b2a2 and e1a2 transcripts in CML patients in Northeast Iran. The mean age in patients with b3a2 positive was younger than b3a2 negative group and neutrophil percentage in the b3a2 and b2a2 positive groups was higher than the b3a2 and b2a2 negative groups. There was no association between e1a2 transcript frequencies, percentage of monocytes and absolute monocytic count in PBS. 
